# P-865. Clinical Characteristics, Risk Factors and Outcome of Critically Ill Immunocompromised Patients with Bloodstream Infections and Sepsis

**DOI:** 10.1093/ofid/ofae631.1056

**Published:** 2025-01-29

**Authors:** Nirusdee Vonineng, Yuda Sutherasan, Jackrapong Bruminhent

**Affiliations:** Faculty of Medicine Ramathibodi Hospital, Mahidol University, Bangkok, Krung Thep, Thailand; Faculty of Medicine Ramathibodi Hospital, Mahidol University, Bangkok, Krung Thep, Thailand; Faculty of Medicine Ramathibodi Hospital, Mahidol University, Bangkok, Krung Thep, Thailand

## Abstract

**Background:**

Immunocompromised patients with sepsis have greater odds of in-hospital mortality compared to immunocompetent patients. A study focused on bloodstream infection (BSI) and sepsis in critically ill immunocompromised (CII) patients has not been extensively explored. We investigated the epidemiology of pathogens, risk factors, outcome, mortality associated with BSI and sepsis among CII patients.

**Methods:**

A retrospective cohort study was conducted at the Medical Intensive Care Unit between January 2022 and December 2023. The participants included patients with immunocompromised status who had suspected sepsis and septic shock and developed documented BSI confirmed by positive blood cultures. A 1:1 propensity score matching without replacement was performed for creating comparable groups and used in Cox proportional hazards modeling to estimate the risk of all-cause mortality within 30 days.

**Results:**

A total of 211 CII patients were included. The mean age (SD) was 61 (16) years, and 57% were male, with a median SOFA score (IQR) of 7 (4-11) and a median APACHE score (IQR) of 16 (14-20). The immunodeficiency profile included hematologic malignancy (37.4%), solid organ malignancy (27.0%), autoimmune disease (19.0%), solid organ transplantation (5.7%), others (10.4%). There were 85 cases (40.28%) had positive blood cultures, predominately showing gram negative rod bacteria (65.9%) focusing of *P. aeruginosa* (17%), *E.coli* (17%) and *K.pneumoniae* (14%). There were 103 (48.82%) patients died within 30 days (p=0.73). There were 85 well balanced matches paired to positive and negative hemoculture groups, respectively. In a propensity score–matched cohort, factors associated with 30-day mortality included a higher SOFA score and lactate level above 4 mmol/L, showing a trend towards increased risk with [HR 1.12 (95%CI, 1.04-1.20, P = 0.003] and [HR 1.91 (95%CI, 1.06-3.42, P=0.031), respectively. Additionally, underlying COPD/asthma appeared to be a protective factor [HR 0.20(95% CI, 0.06-0.66, P = 0.009)].

**Conclusion:**

Among CII patients, BSI, especially those derived from gram-negative bacteria, are not uncommon. Severe conditions characterized by higher SOFA scores and persistently high lactate level from inadequate resuscitation are risk factors for mortality within 30 days.

**Disclosures:**

**All Authors**: No reported disclosures

